# Painful Scars as Pain Generators: A Narrative Review and Case Series of Intralesional Lidocaine Treatment

**DOI:** 10.3390/jcm15218202

**Published:** 2026-10-22

**Authors:** Veronica Gagliardi, Stefano Angiolini, Francesco Ceccherelli, Antonello Lovato, Giuseppe Gagliardi

**Affiliations:** 1 Department of Anaesthesiology and Intensive Care, Venice Hospital, AULSS 3 Serenissima, 30174 Venice, Italy; stefano.angiolini@aulss3.veneto.it (S.A.); giuseppe.gagliardi@aulss3.veneto.it (G.G.); 2 Department of Anaesthesiology and Intensive Care, University of Padua, 35122 Padua, Italy; 3 AIRAS (Associazione Italiana per la Ricerca e l’Aggiornamento Scientifico), Via Nazario Sauro, 29, 36045 Lonigo, Italy; info@ceccherelli.it (F.C.); lovatoantonello@gmail.com (A.L.)

**Keywords:** painful scar, pain generator, lidocaine infiltration

## Abstract

**Introduction**: Active scars can be defined as “pain generators”: the pain associated with them may have an important impact on the patient’s quality of life. During the healing process, several local and systemic neurochemical changes occur, leading to alterations at both the tissue and systemic levels, with changes in the central nervous system (CNS) implicated in the development of chronic pain. Clinically, the features that define a scar as a pathological one include increased firmness, increased tissue thickness, and increased reactivity of the skin after palpation. The therapeutic approach, therefore, includes both local treatments that interfere with structural changes in the scar tissue and systemic treatments. **Materials and Methods**: A critical review of the literature regarding the role of local anesthetics in the clinical management of painful scars has been conducted. The paper also reports a series of cases involving patients affected by painful scars who were successfully treated with local lidocaine injections. A series of five cases of adult patients with active painful scars of post-traumatic and post-surgical etiology were treated with intra-lesional infiltration of 0.5% lidocaine. When appropriate, we associated other techniques. Pain intensity has been assessed using the NRS scale before the start of treatment, at the end of treatment, and at the 3-month follow-up. **Results and Discussion**: Lidocaine infiltration has been associated with a reduction in pain in patients affected by painful scars, as it might reduce the expression of inflammatory mediators. It could also have a neuromodulatory effect by reducing pain signal transduction. Eventually, a key role is the inhibition of NGF binding to its TrkA receptor. In all patients, we observed a long-lasting reduction in pain symptoms, suggesting that changes had occurred in the tissue and in the peripheral nervous system, and a modulation of central nervous system connectivity had been detected. No complications occurred. **Conclusions**: Local treatment with intralesional infiltration with 0.5% lidocaine was associated with clinically meaningful reductions in pain intensity in patients with painful post-traumatic and post-surgical scars, maintaining the benefit on pain over time, with no complications.

## 1. Introduction

### 1.1. The Scar: “A Pain Generator”

Skin or mucosal scars resulting from trauma or surgery can cause pain of varying intensities, which may also involve the autonomic nervous system and the internal organs. The term “pain generator” refers to the various anatomical or functional structures responsible for clinical pain.

Scars can therefore be regarded as “pain generators:, and the pain associated with them can have an important impact on the patient’s quality of life.

The pain resulting from active scars can have several components. Firstly, there is the nociceptive pain component, arising from tissue damage where nociceptors are activated; there may also be a neuropathic pain component, when the somatosensory system is injured; and eventually, a nociplastic pain component may play a pivotal role: it arises from altered nociception involving the activation of peripheral nociceptors without evidence of active tissue damage causing the pain [1]. The estimated prevalence of painful scars is 2% of all scars; in the subgroup of burn scars, it is estimated to be around 30–68% [2].

### 1.2. Physical Examination

The wound-healing process can deviate from the physiological pathway, leading to hypertrophic scars, keloids, and atrophic scars. A hypertrophic scar is characterized by increased secretion of the extracellular matrix, although this is constrained to the area of the initial wound. In a keloid, the scar tissue proliferates beyond the edges of the original wound. These two conditions are regarded as two different stages of the same pathology. An atrophic scar, instead, appears as a depression in the skin, characterized by a deficit of the extracellular matrix [3].

**Figure 1:**
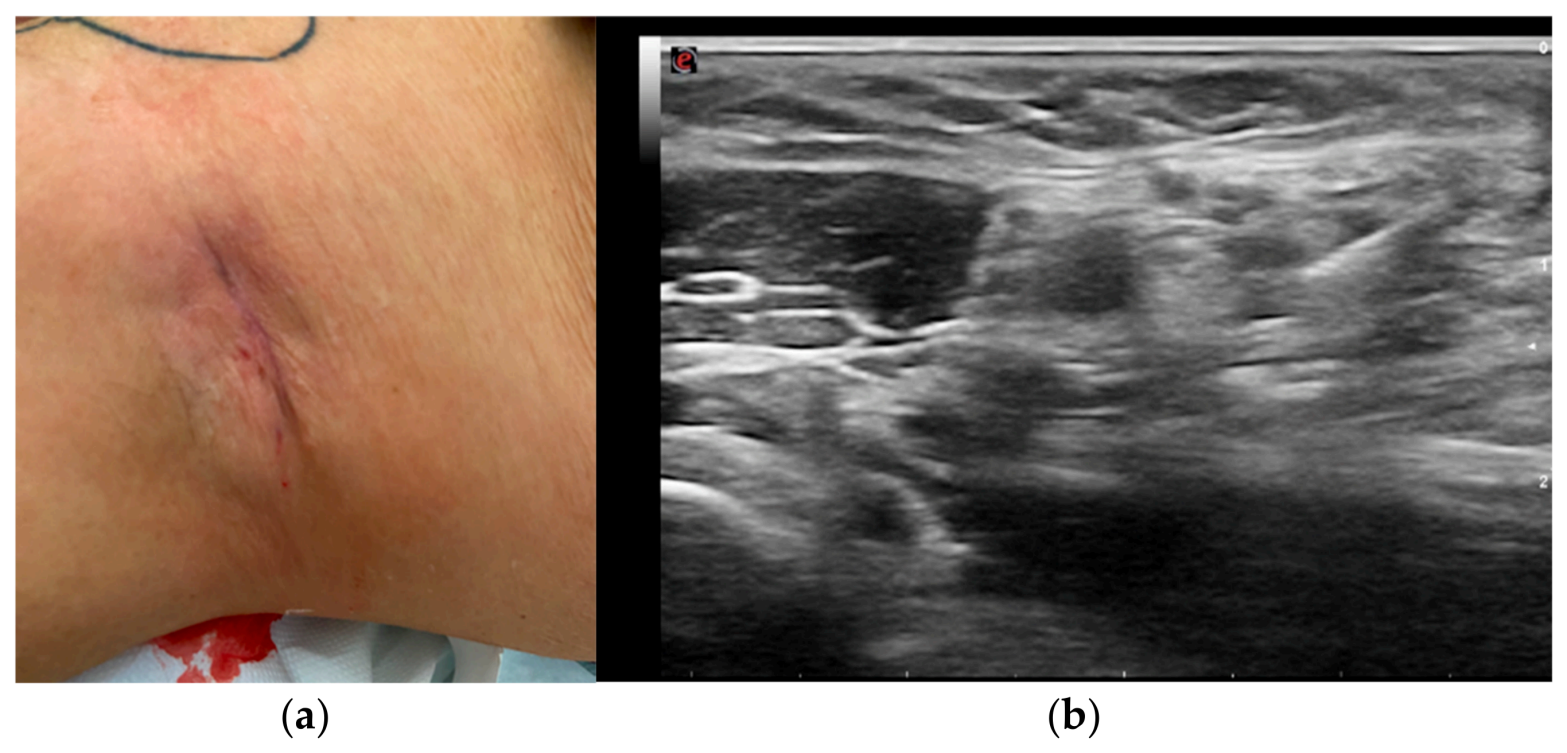
Example of physical and ultrasound examination of the scar. (**a**) Skin scar, wide and adherent, not hypertrophic. (**b**) Ultrasound view of the scar, deep, involving the muscular fascia.

Scars can involve all components of the soft tissues: the skin, the subcutaneous tissue, the superficial and deep fasciae, muscles, and visceral structures [4]. When the dermis and fascia are altered and involved in scarring, their function and ability to interact with the microenvironment are impaired [3]. On physical examination, the findings of a pathological scar—defined as an “active scar”—include increased firmness, increased tissue thickness, and increased reactivity of the skin surface following palpation [4]. Spontaneous pain or pain induced by palpation can also occur. To carry out effective treatment of a painful scar, understanding its extent within the layers beneath the skin and the condition of these tissues is essential [5]. If the scar tissue extends beneath the skin and through the underlying layers, it may cause injury to or entrapment of nerve structures, as well as alterations at the fascial level, profoundly altering the function of entire muscle groups. In such cases, a condition known as a neuroma may develop, in which neurons become trapped within the scar tissue, leading to mechanical compression of the Aδ and C fibers, which causes pain [6]. Both intra-neural and extra-neural structures may be involved in the development of a painful scar; for this reason, a systematic approach to diagnosis is required in order to plan the appropriate treatment. Ultrasound is an important tool for assessing the depth and extent of the scar in different tissues and can also provide information on the involvement of nerve structures and the condition of the connective tissue [7]. It can be routinely used in clinical practice to complete the physical examination [5] (Figure 1).

### 1.3. Pathophysiology of “Active Scars”

Fibrosis and the scar-forming process can alter biomechanics and biochemical signaling, leading to painful symptoms [8,9]. However, neurochemical changes do not occur only in the site affected by the injury, but also at a systemic level: the neurotransmitters released can alter central connectivity and plasticity, a systemic response implicated in the etiology of chronic pain [10]. In particular, after the injury, both free nerve endings and keratinocytes are involved in nociception. Both secrete pro-inflammatory neuropeptides, establishing an inflammatory state that can become chronic on the basis of the pathogenesis of neuropathic pain and central sensitization [11]. Wound healing is a process triggered by inflammatory mediators. Afterwards, mechanical forces guide the remodeling and maturation of the scar by activating mechanoreceptors, which regulate the gene expression of proteins underlying cell proliferation, angiogenesis, and epithelialization. Sometimes, however, the remodeling process is disrupted due to persistent inflammation within the scar. Another factor playing a key regulatory role in scar formation, whether normal or pathological, is the skin’s peripheral sensory and autonomic nervous system. Other neuropeptides, such as VIP, substance P, and CGRP, secreted mainly by the autonomic nervous system (ANS), play a role in modulating metalloproteinases, thereby regulating the granulation tissue and the collagen structure. Inflammatory cytokines, such as TNF-α, IL-1, and IL-6, are secreted by both immune cells and nerve endings [12]. In this context, mast cells also secrete histamine, which plays both a vasoactive role and a role in stimulating fibrosis [12]. Hypertrophic scar tissue exhibits increased expression of Toll-like receptors (epidermal TLR-6 and TLR-7 and dermal TLR-8), the persistent activation of which exacerbates the inflammatory cascade responsible for fibrosis. Finally, during the healing process, calcium plays a key role in intracellular signaling; indeed, the in vitro administration of a calcium antagonist (verapamil) can reduce inflammation and the production of extracellular matrix [13]. In conclusion, pathological fibrosis results from the persistence of the inflammatory response in the tissue, which promotes the proliferation of fibroblasts and the deposition of extracellular matrix by these cells. In this context, dysregulation of TGF-β/Smad signaling is one of the main pathogenic factors. It is a ubiquitous cytokine in the wound-healing process, fundamental in the deposition of the extracellular matrix by regulating scar formation, and can be synthesized by damaged tissues and M2-phenotype macrophages [14]. TGF-β1 also induces angiogenesis via the stimulation of VEGF and PDGF [13]. Furthermore, other essential mediators in the pathogenesis of painful scars are neuropeptides, expressed by autonomic and sensory nerve endings, including calcitonin gene-related peptide (CGRP), substance P (SP), neurokinin A (NKA), and vasoactive intestinal peptide (VIP) [15]; in particular, changes in substance P levels are observed in hypertrophic scars [12]. Neuropeptides modulate wound healing by inducing vasodilation and inflammation, and by stimulating the proliferation of epithelial, vascular, and connective tissues. Moreover, nerve fibers regenerate spontaneously within scars, but their density, morphology, and activity are different from those of healthy tissue; they have a greater density in pathological scars, which consequently exhibit higher concentrations of NPY, VIP, SP, and CGRP compared with healthy tissue, a sign of increased neurogenic inflammation [13,15], the cause of the symptoms of the active scar: pain, itching, allodynia, and altered sensitivity to thermal stimuli [12]. A systemic increase in neuropeptides, particularly CGRP, is also observed, and the process underlies chronic pain [10]. Local neuro-inflammatory overstimulation is probably the result of a spinal reflex arc: tissue damage stimulates C and Aδ fibers, which triggers a central neuro-inflammatory signal leading to increased neuropeptide secretion [3]. In painful scars, in fact, there is an increase in free nerve endings that secrete neuropeptides [6].

#### The Role of Nerve Grow Factor

A mediator that plays a pivotal role in wound healing and is therefore implicated in the pathophysiology of painful scars when dysregulated is NGF (nerve growth factor) [6]. NGF promotes the proliferation of fibroblasts and keratinocytes, the deposition of the extracellular matrix (ECM), angiogenesis, the differentiation of fibroblasts into myofibroblasts, and wound contraction and ECM remodeling [16] following skin tissue damage. Exposed nerve endings release substance P and NKA, which induce the synthesis and release of NGF in keratinocytes; this, in turn, exacerbates the release of vasoactive peptides by the exposed nerve endings, triggering and sustaining neurogenic inflammation [6]. Overexpression of NGF leads to fibrotic tissue abnormalities, whilst downregulation results in delayed wound healing. NGF binds primarily to two receptors, TrkA and p75, which mediate proliferation, survival, and apoptosis through various complex mechanisms during the three phases of wound healing: the inflammatory, proliferative, and remodeling phases. In the first phase, the immune-activating effect on immune cells predominates, promoting the phagocytosis and degranulation of mast cells. In the following stages, NGF induces re-epithelialization, angiogenesis, and neurogenesis, with an anti-apoptotic effect on keratinocytes and neurons, stimulating axonal growth and promoting fibroblast migration. During the remodeling phase, it induces the differentiation of fibroblasts into myofibroblasts and wound contraction. The production of NGF by keratinocytes also increases the expression of α1-adrenergic receptors on free nerve endings, causing pain mediated by sympathetic nervous system activation [17].

### 1.4. Therapeutic Options

In this context, an effective treatment for painful scars is acupuncture, which plays a role in suppressing local and systemic inflammation, stimulating re-epithelialization, and improving microcirculatory function, all of which have a trophic effect by enhancing oxygenation and normalizing pH [18]. Furthermore, micro-needling has been shown to have regenerative effects, inducing the release of trophic and neoangiogenic mediators, normalizing the pattern of collagen organization, and reducing the local inflammatory response [12]. By remodeling the extracellular matrix with other collagen proteins, it also activates the central anti-nociceptive system [19].

Furthermore, acupuncture modulates the peripheral and central nervous systems through the release of endogenous opioids, which have an inhibitory effect on the dorsal horns (by activating inhibitory interneurons), and oxytocin, which inhibits the activity of C-fibers [11].

In cases of chronic pain, the therapeutic approach must include both local treatment that interferes with structural changes within the scar tissue, and systemic treatment, such as the use of drugs with systemic action, including gabapentinoids, sodium channel modulators, ketamine, NSAIDs, opioids, and SNRIs [7]. One systemic approach is auricular acupuncture, which works through the release of β-endorphins, the regulation of pro-inflammatory and anti-inflammatory cytokines, and the activation of the descending anti-nociceptive system [6].

One of the local techniques is the intralesional injection of corticosteroids, which is effective in reducing pain and itching, and acts by inhibiting fibroblast proliferation and promoting collagen degradation [7]. However, it is associated with complications such as atrophy, hyperpigmentation, telangiectasia, ulcers, dermal atrophy, necrosis, and pain during treatment [20].

Other techniques employ electrical neuromodulation: TENS (transcutaneous electrical nerve stimulation), EMS (electrical muscle stimulation), and IFC (interferential current) [7]. Finally, a widely used local approach is the infiltration of the lesion with a local anesthetic, which is an effective technique with long-lasting effects [7]. This treatment is the therapy of choice precisely because of the effect these drugs can have on local changes and on the central processes involved in the development of chronic conditions.

### 1.5. The Role of Local Anesthetics

Thanks to these effects, local anesthetics form the cornerstone of neural therapy, which developed in Europe—primarily in Germany—during the first half of the 20th century; the term “neural therapy” was coined to distinguish the use of these drugs from that of surgical anesthesia. The theory underpinning neural therapy is based on the identification of the so-called “disturbance field”, a region of the body characterized by altered local homeostasis that amplifies to become a systemic pathology. Both the somatosensory system and the autonomic nervous system—in its sympathetic and parasympathetic components—are therefore involved. Local anesthetics form the basis of the therapy, which utilizes their regulatory effect on nerve endings to restore tissue homeostasis. One of the main targets of neural therapy is scar tissue: according to the theory of neural therapy, every scar has the potential to interfere with the sympathetic nervous system, to the extent that it becomes a “disturbing field”. In this context, the infiltration of scar tissue has two effects: the interruption of the afferent stimulus from the disturbance field and a systemic effect on the modulation of sympathetic activity [21,22,23].

## 2. Materials and Methods

This paper presents a critical narrative review of the literature concerning the role of local anesthetics in the clinical management of painful scars and their pharmacological mechanism. Furthermore, a case series of patients with painful scars treated with local lidocaine infiltration is presented, exploiting its role in modulating scar tissue remodeling and the expression of pro- and anti-inflammatory mediators, as well as its ability to modify the neuronal activity underlying the chronicity of pain. A significant improvement in pain symptoms was observed, indicating that lidocaine was effective in silencing the pain generator. All these patients had been previously treated with systemic techniques or other local techniques, which were not effective alone. Nevertheless, we kept the previous treatments, associating them with the intralesional infiltration of lidocaine. We included adult patients affected by chronic pain derived from a painful scar in our group. Informed consent for publication of de-identified data has been acquired from all five patients.

We report five cases of adult patients suffering from painful active scars of post-traumatic and post-surgical etiology, treated with 0.5% lidocaine infiltration; the total dose of lidocaine administered never exceeded 100 mg. We employed a 23 G needle. The injections were performed on the lateral edges of the scar, within the confines of healthy skin, and under the scar to separate it from the underlying tissue. The injection depths depended on the structure involved: it was usually intra-dermal, but it could also be deeper. Sometimes, we employed ultrasound guidance to clarify the size of the scar and to reach the deeper point. The number of injections depended on the size of the scar: the injection points were separated from each other by approximately 1 cm (generally from 2 to 10 cm).

At the physical examination, we identified an active scar as a hyper-reactive scar: it could be spontaneously painful or tender after palpation, hyperalgesic, or hyperemic with increased thickness. After the intralesional infiltration, the pain symptoms related to the scar disappeared [7]. Patients underwent personalized treatment, involving a variable number of sessions, a variable volume of administered anesthetic, and, where appropriate, combined treatment with techniques with a systemic effect. Pain intensity was assessed using the NRS (Numeric Rating Scale). The NRS was administered prior to the start of treatment, at the end of treatment, and at the 3-month follow-up. No complications arose because of the lidocaine infiltration treatment.

## 3. Case Presentation

### 3.1. Case 1: Pain-like Scapulohumeral Periarthritis of the Right Shoulder

Female, 68 years old.

Her past medical history was as follows:

- Previous excision of a dorsal lipoma;
- Arterial hypertension;
- Dyslipidemia.

Medication history: enalapril and atorvastatin.

Diagnostic investigations already performed:

- MRI of the right shoulder: negative.

PAIN HISTORY: For 24 months, pain in the shoulder with functional impairment in abduction and external rotation. NRS at rest: 7.

**Figure 2:**
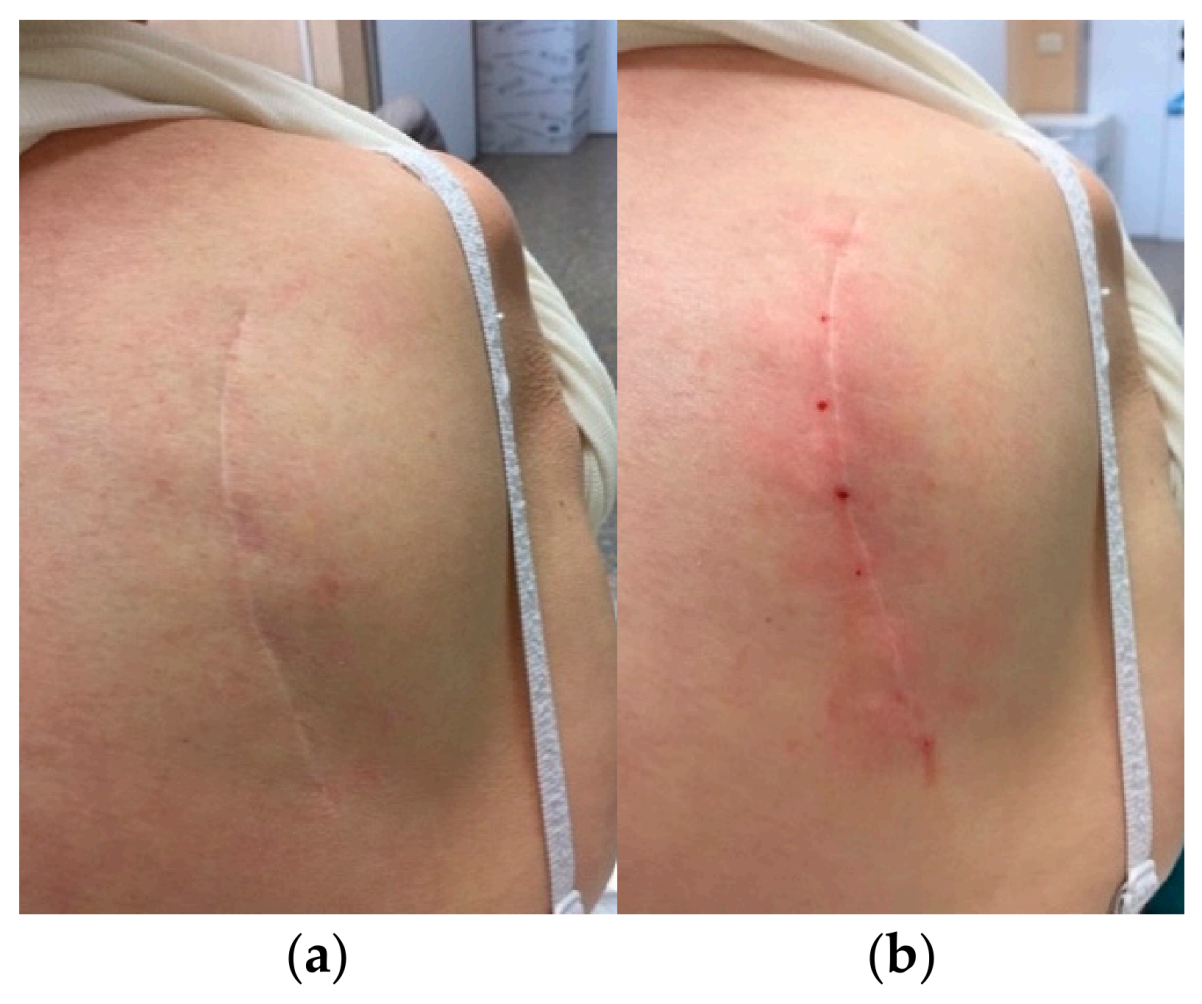
(**a**) Scar at the site of previous lipoma removal. (**b**) After infiltrative treatment.

PHYSICAL EXAMINATION: Whitish, thickened scar, tenaciously adherent to underlying planes, tender on palpation (Figure 2a).

TREATMENT: Five sessions of scar infiltration with 0.5% lidocaine were performed on a weekly basis (Figure 2b).

OUTCOME: At the end of the treatment, complete regression of painful symptoms. NRS: 0; NRS at three months: 0.

### 3.2. Case 2: Pain at the Site of a Previous Hip Prosthesis Wound, Complicated by Infection

79-year-old male.

His past medical history was as follows:

- Lumbar disc herniation;
- Previous right hip replacement, followed by six more revision surgeries at the same site. Complicated by multidrug-resistant *Acinetobacter baumannii* infection.

Medication history: NSAIDs as needed.

PAIN HISTORY: For about 36 months, he has reported pain only at the wound site; present with movement, reduced at rest, NRS: 8. Walks with an aid (cane).

**Figure 3:**
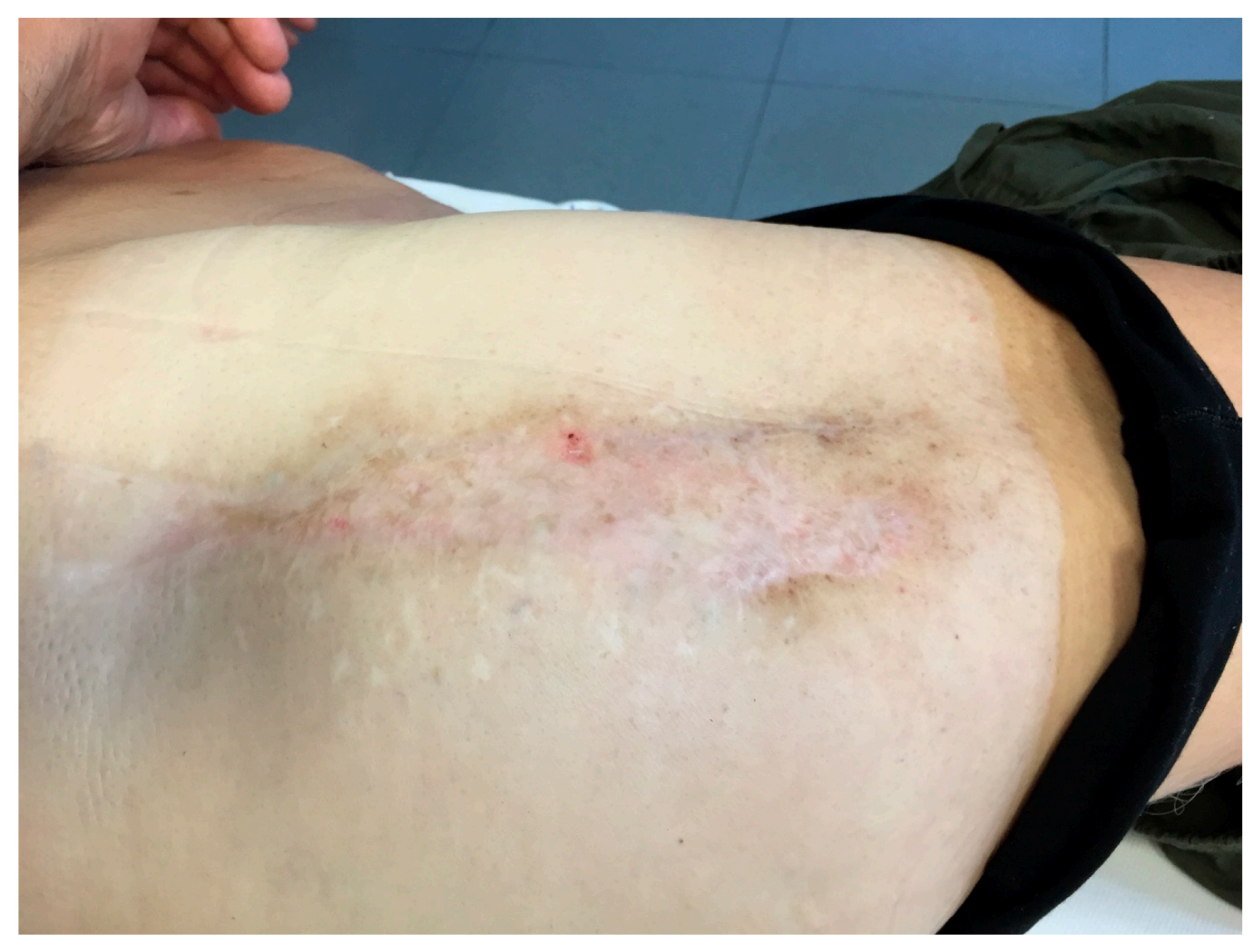
Wide, atrophic scar at the site of a previous surgery complicated by infection.

PHYSICAL EXAMINATION: Irregular scar with hypertrophic and atrophic areas, wide, and firmly adhered to the underlying tissues (Figure 3).

Diagnostic tests already performed:

- Lumbar MRI: negative;
- Scintigraphy: negative for infectious foci.

TREATMENT: Scar infiltrations with 0.5% lidocaine were performed for a total of eight weekly sessions.

OUTCOME: Clinical improvement was already evident after the first session. After six sessions, pain was absent while sitting. After eight sessions, pain was absent when both standing and lying down. NRS: 3 with movement and 0 at rest. Walks without an aid. NRS at three months: 4 with movement, 1 at rest.

### 3.3. Case 3: Pain in the Right Leg with Claudication at the Site of Previous Trauma

75-year-old male.

Past medical history: negative.

Medication history: negative.

Diagnostic tests already performed: none.

PAIN HISTORY: For the past 5 months, pain in the back of the right leg with claudication at 100 m, at the site of a previous wound from an accidental fall (from a ladder). NRS 7.

**Figure 4:**
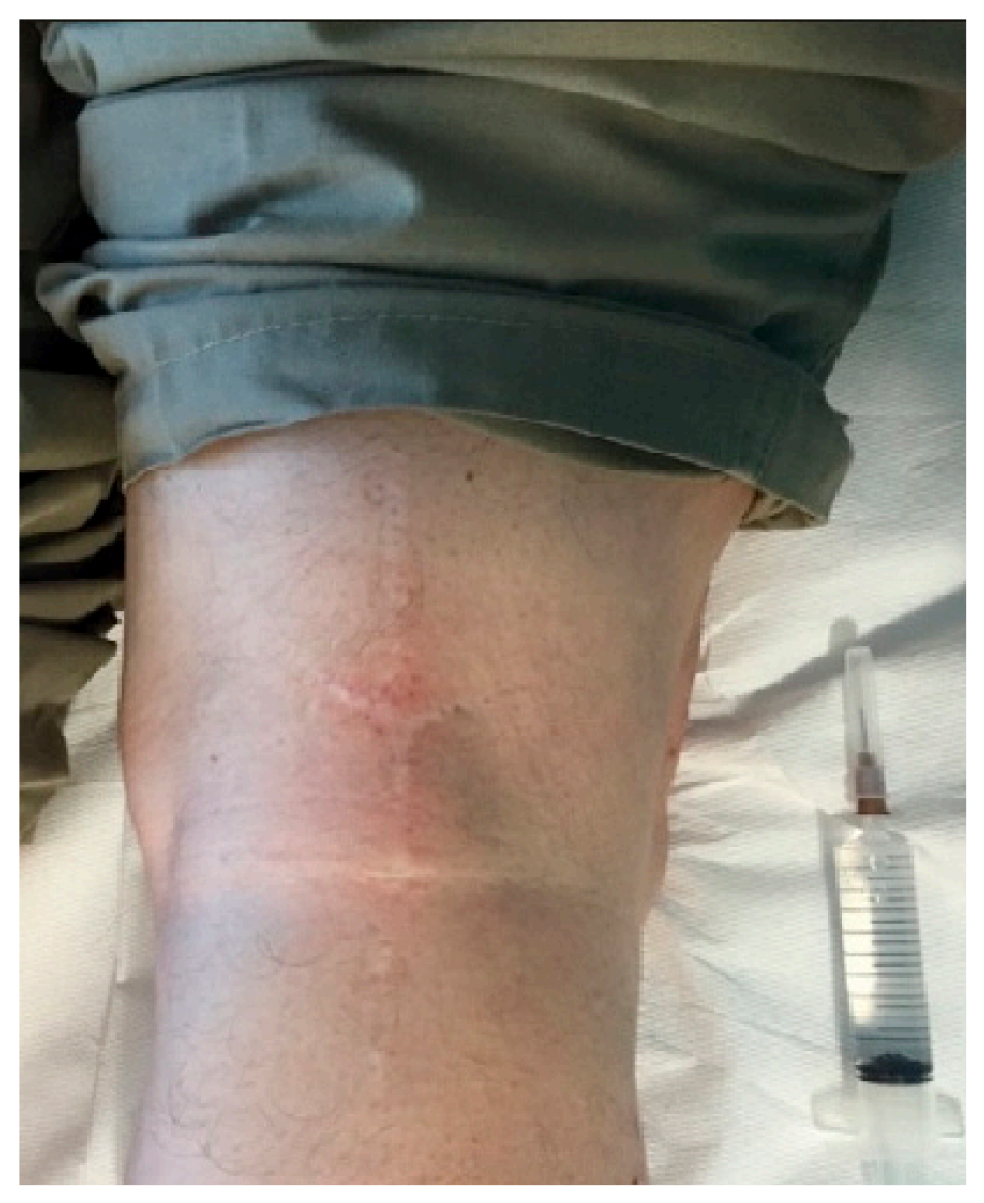
Scar after a fall injury.

PHYSICAL EXAM: Red, raised scar, tender to touch, adherent to deeper layers (Figure 4). TREATMENT: Five sessions of scar infiltration with 0.5% lidocaine were performed weekly. In the first two sessions, only the superficial scar was infiltrated; no improvement was noted. In the following sessions, the deep muscle wound was also infiltrated, resolving the symptoms.

OUTCOME: At the end of treatment, NRS was 0, and normal mobility was restored. NRS at three months: 0.

### 3.4. Case 4: Pain in the Right Hypochondrium with Dysesthesia in the Lower Limbs

30-year-old female.

Past medical history: surgery for dorsal hernia via right thoracotomy.

Medication history: negative.

Diagnostic investigations already performed:

- Spinal cord MRI: mild cord softening at T7 and T8.

PAIN HISTORY: For 7 months, pain in the right hypochondrium with dysesthesia in the lower limbs. NRS: 8.

**Figure 5:**
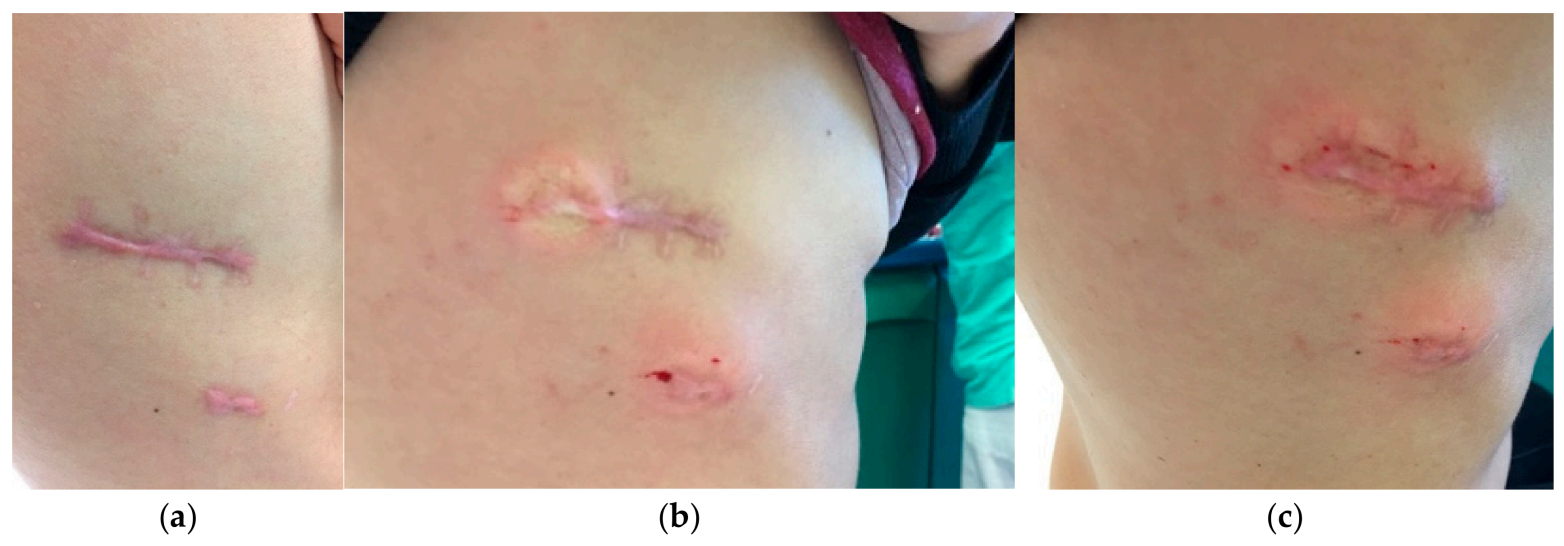
(**a**) Active post-surgical scars. (**b**) Partial intralesional infiltration. (**c**) Complete intralesional infiltration.

PHYSICAL EXAMINATION: The scar is red, thickened, raised, spontaneously painful, and hyperalgesic on palpation (Figure 5a).

TREATMENT: Six sessions of scar infiltration with 0.5% lidocaine were performed on a weekly basis (Figure 5b,c).

OUTCOME: At the end of treatment, NRS was 0, and normal mobility was recovered. NRS at three months was 0.

### 3.5. Case 5: Pain in the Tongue

65-year-old woman.

Past medical history: previous removal of a lingual angioma.

Medication history: none.

Diagnostic investigations already carried out: none.

PAIN HISTORY: Persistent pain at the tip of the tongue for 72 months. NRS score of 8 during activity and 5 at rest.

**Figure 6:**
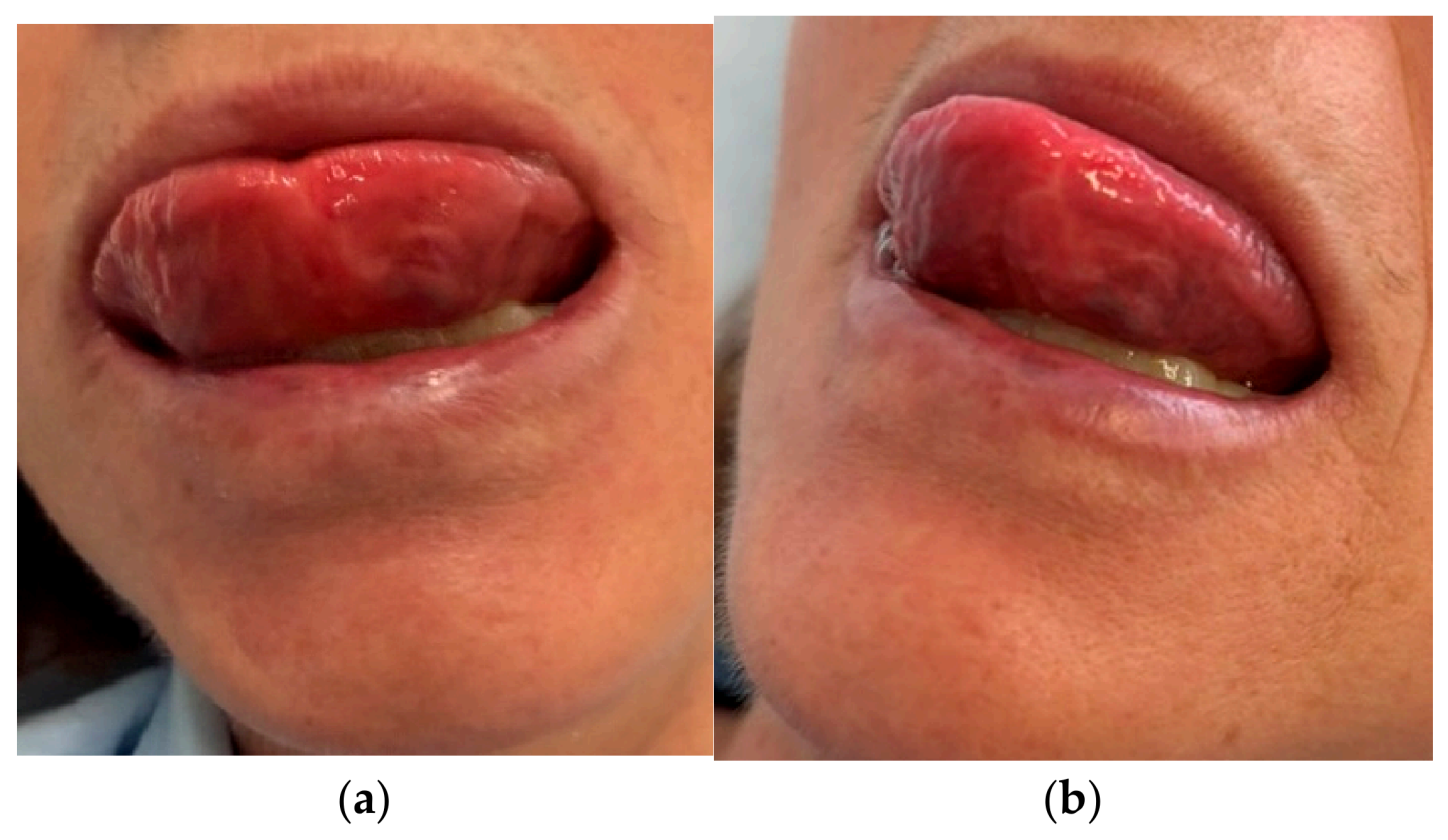
(**a**) Mucosal scar prior to treatment. (**b**) Scar after 5 sessions.

PHYSICAL EXAMINATION: A thickened, whitish scar with increased firmness (Figure 6a).

TREATMENT: Eight sessions of infiltration of the scar with 0.5% lidocaine were carried out at weekly intervals, combined with a block of the sphenopalatine ganglion.

OUTCOME: There was complete remission of pain lasting for several days, with a mean NRS of 1 at rest and 3 during activity (Figure 6b). The mean NRS at three months was 1.

## 4. Results

**Figure 7:**
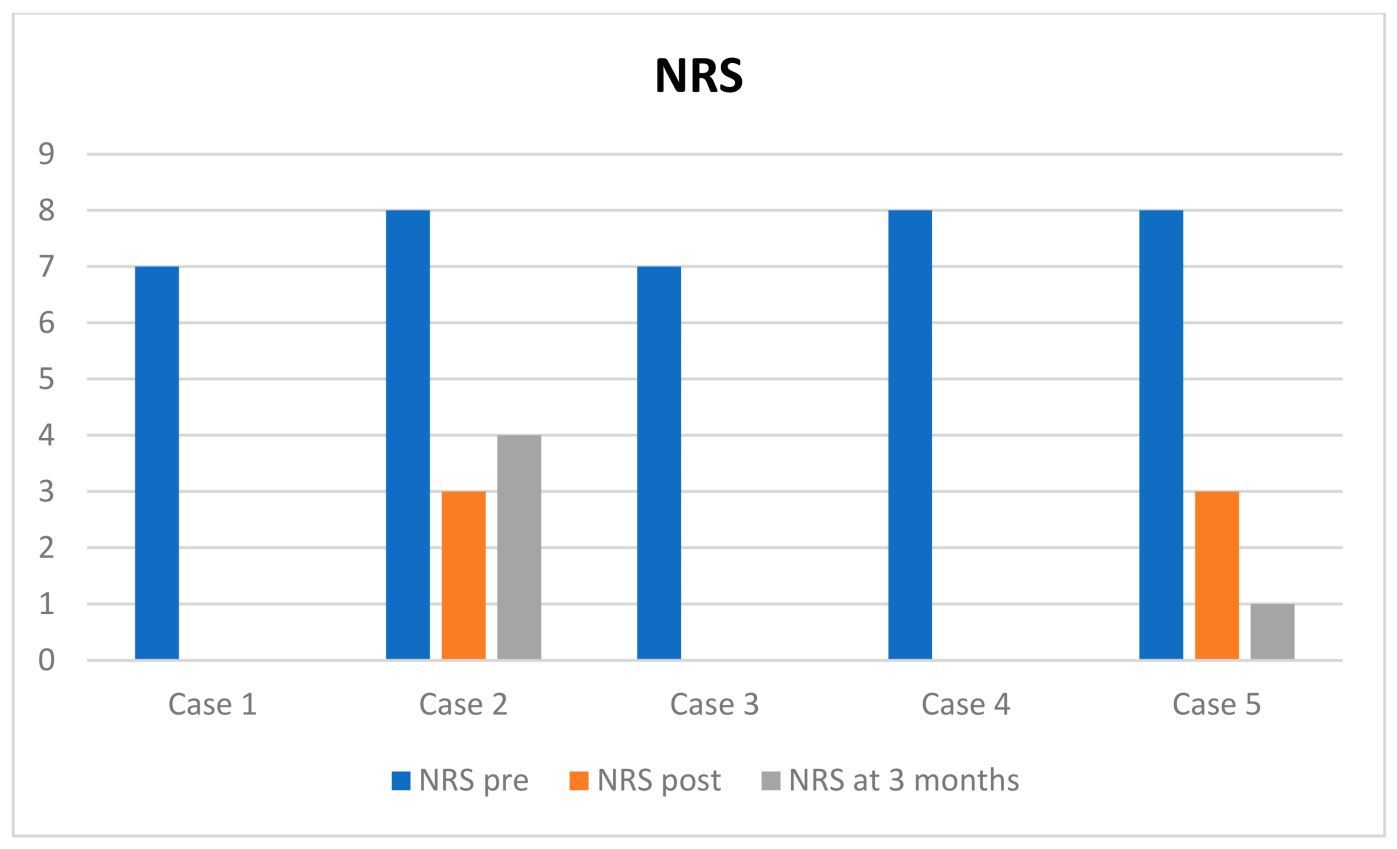
Graph reporting the NRS scores before treatment, after treatment, and at the three-month follow-up.

The results obtained are summarized in Table 1. We observed a reduction in the NRS score in all patients, with values below 5 in all cases at the end of treatment (Figure 7).

## 5. Discussion

Lidocaine infiltration has proved to be effective in the treatment of painful scars because it acts directly on the pathophysiological mechanisms underlying the etiology of the condition. More specifically, lidocaine has been demonstrated to reduce the expression of inflammatory mediators. It is hypothesized that it regulates the expression of vasoactive neuropeptides, thereby alleviating neuropathic pain and neurogenic inflammation by inhibiting the transcription of receptors for chemokines and inflammatory cytokines. Secondly, lidocaine exerts a neuromodulatory effect by reducing pain signal transduction in C-fibers and inhibiting aberrant discharges from damaged neurons, as well as inhibiting sodium channels in spinal cord neurons [24].

Furthermore, at the neuronal level, lidocaine inhibits Gq-coupled receptors by binding to the α subunit, resulting in a reversible and transient downstream effect on neuronal signal transduction, which inhibits cAMP activity and produces an anti-inflammatory effect [25,26].

Eventually, a key regulatory role described for lidocaine is its effect on the binding of NGF to the TrkA receptor: in vitro, a lidocaine concentration of 40–50 mmol inhibits the growth of dendrites in NGF-stimulated neurons.

It has in fact been demonstrated that lidocaine is a competitive ligand for ATP at the TrkA receptor binding site, as the two binding sites of the two molecules have a similar steric structure: the protein sequence of the cytoplasmic site where lidocaine binds the sodium channel is very similar to the one for ATP of the tyrosine kinase receptor activator. Therefore, lidocaine at a concentration greater than 40 mmol inhibits NGF receptor activity, acting as a competitive antagonist. In vivo, a local concentration of 1% lidocaine is considered sufficient to suppress TrkA receptor activation, thereby blocking the pathways responsible for generating chronic pain [27] and inducing a persistent inflammatory state. A 50 mmol/L lidocaine concentration corresponds exactly to 11.72 mg/mL (or grams per liter, g/L), which is equivalent to approximately 1.17%, given the molecular weight of lidocaine, which is approximately 234.34 mg/mmol. We hypothesize that the in vitro mechanism might also have a role in the clinical benefit that the patients experienced.

Despite differences in the number of sessions and whether or not further systemic treatments were combined, local lidocaine infiltration in all patients resulted in a long-lasting reduction in pain symptoms, indicating that a change had occurred at the tissue level and in the activity of the peripheral nervous system at the site, and we hypothesized that a modulation of central nervous system connectivity might be implicated.

## 6. Conclusions

In conclusion, in this case series, intralesional 0.5% lidocaine was associated with clinically meaningful reductions in pain intensity in patients with painful post-traumatic and post-surgical scars. The findings support the feasibility of this approach and provide a rationale for further investigation of the potential peripheral and neuro-inflammatory mechanisms involved. However, because of the small sample size, heterogeneous clinical presentations, concomitant interventions, and absence of a control group, these observations cannot establish treatment efficacy or causality. Prospective controlled studies with standardized injection protocols, validated functional and pain outcomes, and mechanistic assessments are warranted. In our patients, the intralesional infiltration provided sustained relief from pain symptoms over time, with no complications. From a biochemical perspective, lidocaine plays a key role in disrupting the intercellular crosstalk that sustains local inflammation and in inhibiting the neuronal signaling underlying the development of chronic pain. To confirm these findings, further studies involving a larger sample size are needed, as well as studies comparing these results with systemic and other local techniques.

## Figures and Tables

**Table 1:** Case features: age, sex, duration of pain, any associated systemic treatment, number of sessions, pre-treatment NRS, post-treatment NRS, NRS at 3 months, and complications.

Reference	Age	Sex	Duration of Pain (Months)	Associated Systemic Treatment	Number of Therapy Sessions	NRS (Pre)	NRS (Post)	NRS at 3 Months	Complications
Case 1	68	F	24	none	5	7	0	0	None
Case 2	79	M	36	FANS	8	8	3	4	None
Case 3	75	M	5	none	5	7	0	0	None
Case 4	30	F	7	none	8	8	0	0	None
Case 5	65	F	72	block of the sphenopalatine ganglion	8	8	3	1	None

## Data Availability

The original contributions presented in this study are included in the article. Further inquiries can be directed to the corresponding author.

## Abbreviations

The following abbreviations are used in this manuscript:

NGF	Nerve growth factor
TrKA	Tyrosine kinase A
VIP	Vasoactive Intestinal Peptide
CGRP	Calcitonin Gene-Related Peptide
TNF	Tumor necrosis factor
IL-1	Interleukin 1
IL-6	Interleukin 6
TLR	Toll-like receptor
TGF	Transforming Growth Factor
VEGF	Vascular Endothelial Growth Factor
PDGF	Platelet-Derived Growth Factor
NKA	Neurokinin A
NPY	Neuropeptide Y
ECM	Extracellular matrix
NSAIDs	Non-steroidal anti-inflammatory drugs
SNRI	Serotonin and Norepinephrine Reuptake Inhibitors
TENS	Transcutaneous Electrical Nerve Stimulation
EMS	Electrical Muscle Stimulation
IFC	Interferential Current
NRS	Numerical rating scale
